# Low-Molecular-Weight NGF Mimetic Corrects the Cognitive Deficit and Depression-like Behavior in Experimental Diabetes

**Published:** 2017

**Authors:** R. U. Ostrovskaya, S. S. Yagubova, T. A. Gudasheva, S. B. Seredenin

**Affiliations:** V.V. Zakusov Institute of Pharmacology, Baltijskaya Str., 8, Moscow, 125315, Russia

**Keywords:** depression, diabetes, dipeptide NGF mimetic, learning

## Abstract

Based on the comorbidity of diabetes, depression, and dementia and recognizing that a deficiency of the nerve growth factor (NGF) is involved in all of these kinds of pathologies, we studied the effect of the mimetic of dimeric dipeptide NGF loop 4, GK-2, on a model of streptozotocin-induced type 2 diabetes in C57Bl/6 mice. GK-2 [hexamethylenediamide bis-(N-monosuccinyl-glutamyl-lysine)] was synthesized at the V.V. Zakusov Scientific Research Institute of Pharmacology. The study revealed the ability of GK-2 to ameliorate hyperglycemia induced by streptozotocine (STZ 100 mg/kg i.p.) in C57Bl/6 mice, to restore learning ability in the Morris Water Maze test, and to overcome depression after both intraperitoneal (0.5 mg/kg) and peroral (5 mg/kg) long-term administration. The presence of the listed properties and their preservation in the case of peroral treatment determines the prospects of research. Taking into account the previous findings on the ability of GK-2 to selectively activate PI3K/Akt, these data suggest that Akt-signaling is sufficient for pancreatic beta cell function. GK-2 has been shown to exhibit pronounced neuroprotective activity. The coexistence of neuroprotective and antidiabetic effects is in agreement with the fundamental concept holding that the function of neurons and pancreatic beta cells is controlled by similar mechanisms.

## INTRODUCTION

In the several decades that have elapsed since it was discovered that neurotrophic factors play a key role in the development and maintenance of the viability of neurons [1], facts showing that they exhibit a similar regulatory activity at the level of non-neuronal systems have been obtained [2]. An understanding of the role of neurotrophins in the development of pancreatic β-cells was one of the important results of these discoveries. The data provide grounds to believe that the similarity between the growth factors and differentiation is responsible for the similarity between pancreatic β-cells and neurons, which form via the same fundamental development program, although they originate from different cell lineages [3]. The regulatory role of neurotrophins in pancreatic β-cells has been confirmed in a number of studies [4, 5]. The effect of the nerve growth factor (NGF) on pancreatic β-cells was found to be mediated by TrkA, the high-affinity neurotrophin receptor [6]. NGF ensures β-cell neogenesis not only during the fetal and neonatal periods, but also in adult organisms [7]. The removal of NGF from a β-cell culture medium [8] and administration of antibodies against this neurotrophic factor [9] enhances β-cell apoptosis. Convincing evidence has been obtained showing that a reduced NGF level in type 2 diabetes mellitus (T2DM) decreases the proliferation of and/or enhances β-cell apoptosis [10-12].

Meanwhile, the comorbidity of T2D and cognitive deficit (reduced information-processing speed, reduced verbal memory and conceptualization), whose risk in T2DM is much higher than in healthy individuals, is well-known. According to epidemiological data, the degree of increase in risk ranges between 50 and 150% [13, 14]. Post-mortem studies have revealed a decreased NGF level in the frontal cortex of patients in the phase that precedes Alzheimer’s disease (AD) [15]. A reduced activity of choline acetyltransferase, the enzyme whose activity in cholinergic neurons of the basal brain structures is regulated by NGF, is already in fact in this phase. It has been demonstrated that the level of TrkA receptors in the hippocampus, the brain structure responsible for the main cognitive functions and memory, in particular, is reduced in patients with mild cognitive impairment [16]. Hippocampal atrophy is an important prognostic sign of an aggravation of the cognitive pathology and a transition from mild cognitive impairment to AD [17]. Deficiency in NGF plays an important role in it, since this neurotrophin prevents the formation of β-amyloid peptide (Aβ1–42) [18]. The decrease in the NGF level accompanying a cognitive deficit is associated with an increased level of its precursor (proNGF) that suppresses the proliferation and differentiation of the basal brain and hippocampal structures [19]. A shift in the proNGF/NGF ratio towards precursor prevalence is regarded as the main reason for cholinergic deficit, leading to cognitive impairment [20].

The risk of depression and depressive-like behavior in T2DM is at least twice as high as that in individuals without resistance to insulin [21]. The bilateral comorbidity of these disorders (depression aggravates the course of diabetes and vice versa) has been studied [22, 23]. In addition to the convincing data on the role of a deficiency in the brain-derived neurotrophic factor (BDNF) in the pathogenesis of depressive states of different etiologies, including in patients with diabetes [24], it has been demonstrated that the activity of NGF drops both in depression and in diabetes, which is considered to be an important factor that determines their comorbidity. A meta-analysis of 21 publications [25] confirmed a statistically significant decrease in the blood level of NGF in depression, which correlated with impairment intensity. It has been suggested that the reduced level of NGF in blood serum should be regarded as a biomarker for major depression [26]. Such a reduction is also observed in patients with bipolar disorder [27] and senile depressions [28]. Post-mortem examinations of brain tissues from suicide victims have revealed an almost twofold decrease in NGF expression and a more than threefold decrease in TrkA density [29].

A combination of the reported data demonstrates that NGF could be used in patients with type 2 diabetes mellitus because of its ability to maintain β-cell function, stimulate insulin secretion, and simultaneously impede the development of diabetes mellitus and its comorbidities. However, in their attempts to use native NGF, researchers have faced a problem associated with the unsatisfactory pharmacokinetic properties of this protein molecule (low biological stability and inability to pass through biological barriers when administered systemically) and pleiotropicity of NGF activity, which may result in such side effects as weight loss and hyperalgesia. Meanwhile, the effectiveness of topical administration of NGF in trophic ulcers of diabetic genesis has been reported [30]. As for systemic administration of NGF, phase I/II clinical trials of recombinant NGF have revealed a tendency towards a favorable effect in patients with diabetic neuropathy; however, side effects and the lack of a therapeutic effect were observed when a broader patient population was used in phase III trials [31].

One of the strategies used to overcome the drawbacks of native neurotrophins involves the design of low-molecular-weight agents that can induce NGF-like therapeutic effects upon systemic administration without the side effects typical of native NGF. Several compounds of this type have been reported; in particular, NGF mimetic of nonpeptide structure, compound MT-2 [32], and peptide NGF-mimetic BB14 [33, 34]. However, the effects of these compounds have been studied only in *in vitro *systems.

A dimeric dipeptide NGF mimetic GK-2 (hexamethylenediamide-*bis*-(N-monosuccinyl-glutamyl-lysine)) has been designed at the V.V. Zakusov Research Institute of Pharmacology on the basis of the structure of the NGF loop 4 β-turn. It exhibited a high neuroprotective activity in *in vitro *experiments, as well as *in vivo *in models of stroke, Alzheimer’s and Parkinson’s diseases, and had none of the side effects typical of native NGF. GK-2 was shown to activate TrkA receptors [35-37].

Preliminary experiments in rats demonstrated that GK-2 exhibits anti-hyperglycemic activity [38]. On the basis of the comorbidity of diabetes and cognitive impairment and depression, we modeled streptozotocin-induced diabetes in mice and studied the effect of GK-2, the original NGF mimetic, on the cognitive impairment and depressive-like behavior in these animals.

## EXPERIMENTAL

**Animals**

Male C57Bl/6 mice with an initial body weight of 23 – 28 g purchased from the Stolbovaya breeding farm were used in the experiments. The animals were kept under standard vivarium conditions, with unrestricted access to food (except for 16 h prior to streptozotocin administration) and water. The guidelines for ethical rules in the care and use of animals in research summarized in the European Communities Council Directive 86/609/EEC were followed.

**Experiment design**

Type 2 diabetes mellitus was induced by intraperitoneal (i.p.) administration of streptozotocin (STZ, Sigma, USA) at a dose of 100 mg/kg, which was effective for C57Bl/6 mice [39].

The mice were randomly divided into four groups: group 1 (passive control, *n *= 10), group 2 (active control, *n *= 11), and experimental groups 3 (*n *= 11) and 4 (*n *= 12). The mice in the passive control group received saline, either i.p., or perorally (*per os*), for 31 days. No significant differences between i.p. or per os administration of saline during the entire experiment were revealed, so these animals were merged into one group. ;The animals from the active control group received saline i.p. for 14 days; a single dose of STZ (100 mg/kg) was administered i.p. on day 15 after 16-hour fasting; then, mice continued to receive saline for 16 days.

The low molecular weight (831 Da) of GK-2 makes it reasonable to study the effects of both i.p. and the peroral route of administration. The effect of GK-2 administered *per os *needs to be studied, since this compound is intended to be used as a drug for long-term clinical application. The freshly prepared GK-2 solution (in 0.9% NaCl) was administered once a day during 14 days: in study group 3, i.p. at a dose of 0.5 mg/kg; in study group 4, *per os *at a dose of 5 mg/kg. On day 15 (30 min after the animals had received the final dose of GK-2), they were i.p. treated with STZ (100 mg/kg) on an empty stomach; then, both groups of mice continued to receive GK-2 for 16 days.

The glucose level in the blood collected from the tail vein was measured using a One Touch Ultra glucometer (USA). The dynamics of the effect of GK-2 was assessed using the indicator of relative antihyperglycemic activity (Ag) according to the formula:

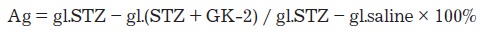



where gl.STZ is the blood glucose level in the active control group (group 2); gl.STZ + GK-2 is the blood glucose level in the study group 3 or 4; and gl.saline is the blood glucose level in the passive control group (group 1).

**Studying the effect of GK-2 on learning ability in the Morris water maze**

Spatial learning and memory were assessed 24 h after the mice had received the final dose of GK-2 (day 17 after administration of STZ) using the Morris water maze [40]. The experimental device consisted of a pool 150 cm in diameter with 60-cm-high walls filled with water (23–25°C). The pool was imaginatively divided into four quadrants. A platform 9 cm in diameter, 1 cm higher than the water level, was placed in the center of one quadrant.

During day 1, the animals were allowed to find the visible platform. If the mouse did not find the platform during the 60 s cut-off, it was placed on the platform and allowed to stay there for 20 s before returning to its home cage. Four trials (one per each quadrant) were used. After 24 h, a platform sub merged 1 cm below the water level was placed onto the same spot as in day 1, but water was preliminarily whitened with milk. Identically to day 1, four trials were used, one for each quadrant. The same procedure was repeated on days 3, 4, 5, and 8. The number of animals that found the platform within the 60 s cut-off was recorded.

**Studying the effect of GK-2 using the depression model**

The depressive-like behavior (the behavioral despair) was assessed using the modified forced swim test on days 45 and 46 after discontinuation of GK-2 [41, 42]. Cylindrically shaped vessels 10 cm in diameter and 30 cm high (OOO Research and Production Company Open Science) were filled with water (23–25°C) to the level of 20 cm from the bottom. On day 1, the animal was placed into the vessel for 10 min and its behavior was video-recorded in the interval between the 2^nd^ and the 6th minute. The test was repeated for 6 min after 24 h. Active swimming and immobilization durations in both sessions were determined using the RealTimer software. According to the definition given by the authors of the test, active swimming implied the periods when the forelimbs moved upward along the cylinder walls, while immobilization implied remaining completely motionless or making the minor movements necessary to maintain the head above water. The total duration of immobilization episodes was the key parameter of the severity of depressive-like behavior in this test.

Exploratory behavior, as well as the general locomotor activity, was assessed using the open field test 2 days prior to performing the Morris water maze. The animals were placed in the center of the open field, and the horizontal motor activity and the numbers of holes and vertical bars were measured during 5 min.

The animals’ body weight was measured every 3 days.

**Fig. 1:**
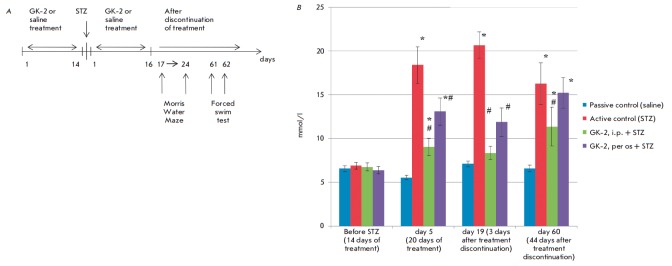
Design of the experiment (*A*) and the dynamics of the blood glucose level (mmol/l) in C57Bl/6 mice (*B*) in the following groups: passive control (Saline + Saline), active control (Saline + STZ 100 mg/kg, i.p. + Saline), GK-2 treated group 3 (GK-2 0.5 mg/kg, i.p. + STZ 100 mg/kg, i.p. + GK-2 0.5 mg/kg, i.p.), GK-2 treated group 4 (GK-2 5 mg/kg, per os + STZ 100 mg/kg, i.p. + GK-2 5 mg/kg, per os). Data are presented as M ± SEM. The statistical significance of the differences was calculated using the Mann–Whitney U-test: * p < 0.05 compared to passive control; # p < 0.05 compared to active control (STZ).

*Fig. 1A* shows the order in which the compounds were administered and behavioral tests were performed.

**Statistical analysis**

The experimental data are shown as mean values, with the mean error and the standard error of the mean (M ± SEM) indicated. The statistical analysis was performed using the Statistica 8.0 software. The statistical significance of intergroup differences was assessed using the nonparametric method, the Mann–Whitney U test. The χ^2^ test was used for the parameters measured in %. The results were considered to be statistically significant at *p *≤ 0.05.

## RESULTS

Data on the dynamics of the blood glucose level in different groups are presented in *Fig. 1B*. While the glucose level in the peripheral blood of mice in the passive control group was 6–7 mmol/l, administration of STZ at a dose of 100 mg/kg to C57Bl/6 mice increased that blood glucose level to 16–20 mmol/l, which is close to the values obtained earlier in the experiments with rats [38]. In full compliance with the antihyperglycemic effect of GK-2 observed in the experiments with rats, we revealed the antihyperglycemic effect of GK-2 in mice. It is important to emphasize that the antihyperglycemic effects were similar for rats and mice: e.g., the calculated Ag parameter on day 17 after administration of STZ to rats was 80%, being 90% on day 19 in mice.

Assessment of the cognitive function performed 24 h after the final dose of GK-2 had been injected demonstrated (*Table*) that, whereas the number of animals that found the platform within 60 s in repeated tests significantly increased in the passive control group, this occurred muchmore slowly in the active control group (the differences between the two groups were statistically significant on days 4 and 8). These results agree with the data on cognitive impairment in STZ-induced diabetes [43]. Intraperitoneal administration of GK-2 caused a statistically significant increase in the number of animals that found the platform on days 2, 4, and 8 of training compared to the animals in the active control group. Upon administration *per os*, the learning ability significantly increased only on test day 2. It should be mentioned that in the beginning of the experiment, the learning ability of mice for both administration routes was even higher than that in the passive control group. The intergroup differences were significant on test days 3 and 5 as well (except for day 5 in the group that received GK-2 *per os*, when the differences between the active control and the study group failed to reach the level of statistical significance).

**Table:** Learning ability of mice in the Morris water maze (the percentage of animals that found the platform within the 60 s cut-off time)

Group	day 2	day 4	day 8
Group 1 Passive control (saline)	14.3%	85.7%	100%
Group 2 Active control (STZ, 100 mg/kg)	9.09%	54.54%*	72.7%*
Group 3 GK-2, 0.5 mg/kg i.p. + STZ	27.3%*#	72.7%*#	90.9%*#
Group 4 GK-2, 5 mg/kg per os + STZ	50%*#	50%*	100%#

The statistical significance of differences was assessed using the χ^2^ test.

^*^ *p* < 0.05 compared to passive control group (saline).

^#^ *p* < 0.05 compared to active control group (STZ).

The effect of GK-2 on the severity of the depression-like behavior was assessed in a long-term period after STZ administration (day 45), since the duration of the depressive-like behavior in the diabetes model was reported to be rather long [25].

**Fig. 2:**
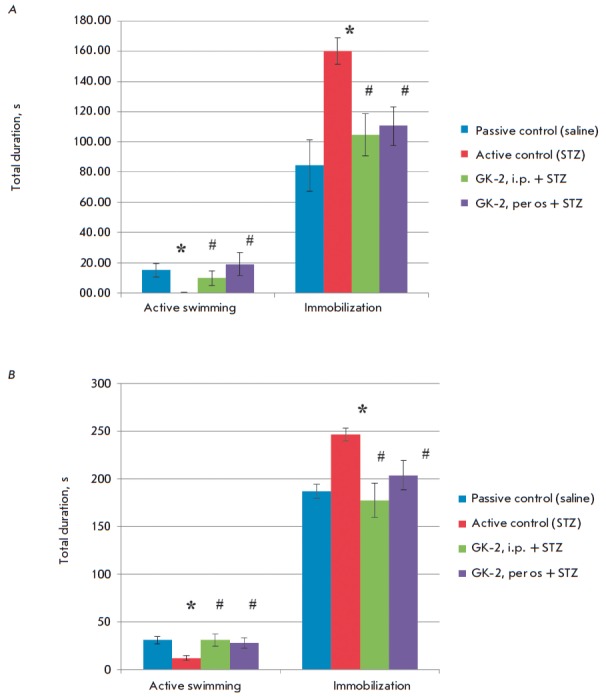
Indicators of a depressive-like status in C57Bl/6 mice: the total duration of active swimming and immobilization (s) on days 61 (A) and 62 (B) after STZ administration. The groups and statistical data are similar to those in *Fig. 1B*

Comparison of active swim test parameters and the immobilization duration in different groups revealed the following regularities (*Fig. 2*): In mice in the active control group, immobilization duration increased, while the duration of active swimming decreased compared to the parameters in the passive control group, while i.p. administration of GK-2 reduced the immobilization duration and increased the active swimming duration, making them as high as the control values. The intensity of the effect of GK-2 administered *per os *was the same as upon i.p. administration.

Similar regularities were observed on day 2: increased immobilization duration and reduced active swimming duration in the active control group, where GK-2 reduced the severity of depression when administered both i.p. and *per os*.

In order to interpret the results, we needed to understand whether the streptozotocin-induced behavioral disorders were related to the overall wellbeing of the animals (reduced motor activity and body weight loss). In order to answer this question, we performed the open field test 2 days prior to the Morris water maze, where changes in neither the orientational nor exploratory activity and overall mobility were observed in the animals treated with STZ. GK-2 upon both administration routes had no effect on these indicators. It was demonstrated that, unlike the passive control group where animal body weight increased during the entire experiment (10.5% with respect to the initial weight by the time the Morris water maze was performed and 16.7% by the time the forced swim test was formed), a slight decrease in body weight by the time of Morris water maze study (–6.7%) and body weight gain by the time of the forced swim test (1.8%) were observed in the active control group. GK-2 reduced this effect of STZ administered both i.p. (–2 and 4.6%, respectively) and *per os *(1 and 10%, respectively). Therefore, the resulting data allow one to rule out the changes in the overall wellbeing of animals as the reason for the STZ-induced behavioral disorders and their normalization due to the administration of NGF mimetic.

## DISCUSSION

**Fig. 3:**
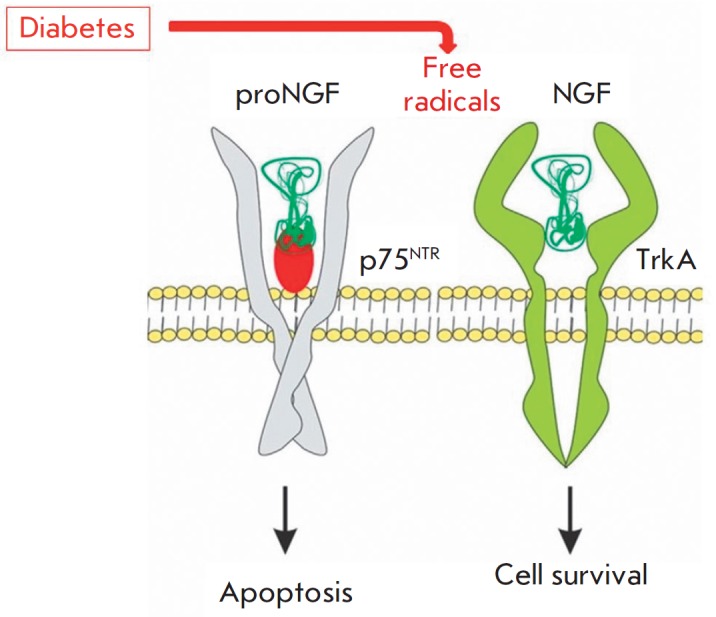
NGF is synthesized from the precursor, pro-NGF. NGF binds to the TrkA receptor and this interaction induces the activation of the signaling pathway of β-cell survival. Diabetes-induced hyperglycemia is known to cause the oxidative stress that decreases protease activity, thus provoking pro-NGF accumulation resulting in β-cell apoptosis (modified from [19, 48])

We reproduced the known model of diabetes mellitus with specific behavioral signs [25, 43] and described for the first time the ability of GK-2, the low-molecular-weight mimetic of the nerve growth factor, to eliminate these behavioral disorders. The main role in the development of a deficiency of NGF in diabetes is known to be its reduced formation from the proNGF precursor as a result of hyperglycemia-induced oxidative stress [44, 45], which suppresses protease activity and shifts the proNGF/NGF ratio towards the precursor prevalence that promotes apoptosis of insulin-secreting cells, contrary to mature NGF, exerting an antiapoptotic effect (*Fig. 3*).

Streptozotocin facilitates free radical formation and alkylates DNA [46]. Administration of STZ reproduces not only the reduced NGF level typical of diabetes [47], but also the increased proNGF level [48]. It has been experimentally demonstrated that the degrees to which the proNGF level increases and mature NGF and phosphorylated TrkA receptors decrease correlate with the severity of the cognitive impairment [49]. The shift in the proNGF/NGF ratio towards the precursor is considered to be the main reason behind the cholinergic deficit that causes cognitive impairment [20].

Identically to the native NGF molecule, GK-2 activates TrkA receptors and alleviates the toxic effects of H_2_O_2_ [35]. In addition, it reduces the blood level of malonic dialdehyde in diabetic mice [50]. An assumption can be derived from these data that the antihyperglycemic effect of GK-2 is caused both by its direct effect on NGF receptors and by its ability to eliminate the toxic effect of free radicals, which can normalize the formation of NGF from its precursor.

We experimentally reproduced the main metabolic effect of STZ – the hyperglycemic effect – and also its behavioral effects imitating the behavioral disorders in diabetic patients: namely, cognitive impairment [14, 16] and development of a depressive-like behavior [51-53]. The ability of GK-2 to attenuate the severity of the cognitive deficit accompanying a diabetes model was revealed. This fact agrees with the positive cognitive effect of GK-2 observed in the Alzheimer’s disease model [54]. The antidepressant effect of GK-2 was described for the first time. The combination of the antidiabetic and antidepressant activities of GK-2 is especially important, because conventional antidepressants not only do not attenuate diabetes signs, but can also increase the risk of its development [55].

It is important to emphasize that the activity of GK-2 is maintained in the case of peroral administration, which is a requisite for drugs used to treat chronic conditions. The combination of the antidiabetic activity of GK-2 with its long-term positive effect on the It is important to emphasize that the activity of GK-2 is maintained in the case of peroral administration, which is a requisite for drugs used to treat chronic conditions. The combination of the antidiabetic activity of GK-2 with its long-term positive effect on the cognitive function and antidepressant properties is an important additional characteristic of this compound. GK-2 is intended for use in the therapy of post-stroke sequelae, since it is known that stroke and diabetes are comorbid and that there is a high rate of development of cognitive deficit and depressive disorders during the post-stroke period [56] .

It has been demonstrated previously [57] that the NGF mimetic GK-2 selectively activates only one of the two main signaling pathways, the PI3K/Akt pathway involved in the neuroprotective effects of neurotrophins, by activating TrkA [58]. The data on the antidiabetic activity of GK-2 allow one to suggest that Akt signalization is sufficient to maintain the β-cell function. The significance of these data mainly consists in the fact that they can lead to new concepts of diabetes development mechanisms and could serve as a basis for the design of antidiabetic agents that exhibit cytoprotection of β-cells. The combination of the neuroprotective and antidiabetic effects of GK-2 is consistent with the earlier stated fundamental concept that the mechanisms of regulation of the function of neurons and pancreatic β-cells are similar [59] and the subsequent conclusion about the reasonability of studying the potential antidiabetic properties of neuroprotective agents that eliminate the deficiency of neurotrophic factors [60].

## CONCLUSIONS

The hyperglycemic, amnestic, and depressive-like effects of STZ were reproduced in this study. The ability of GK-2, the dimeric analog of nerve growth factor loop 4, to have an antihyperglycemic effect and attenuate the severity of the cognitive deficit that develops in a diabetes model has been revealed. The anti-depressant activity of the compound has been established for the first time. Further development of GK-2 is promising due to its combination of antidiabetic activity and positive effect on cognitive functions, as well as antidepressant properties and maintenance of activity when administered *per os*.

In view of the data on the pronounced neuroprotective activity of GK-2 previously obtained at the Research Institute of Pharmacology, the antidiabetic activity of this compound can be regarded as an important argument in support of the fundamental concept that the function of neurons and pancreatic β-cells is controlled by similar mechanisms.

## Abbreviations

AD: Alzheimer’s disease
BDNF: brain-derived neurotrophic factor
i.p.: intraperitoneal
NGF: nerve growth factor
per os: peroral
STZ: streptozotocin
T2D: type 2 diabetes
